# Fuzzy Logic-Based Risk Assessment of a Parallel Robot for Elbow and Wrist Rehabilitation

**DOI:** 10.3390/ijerph17020654

**Published:** 2020-01-19

**Authors:** Paul Tucan, Bogdan Gherman, Kinga Major, Calin Vaida, Zoltan Major, Nicolae Plitea, Giuseppe Carbone, Doina Pisla

**Affiliations:** 1 CESTER, Technical University of Cluj-Napoca, 400641 Cluj-Napoca, Romania; paul.tucan@mep.utcluj.ro (P.T.); majorkinga@yahoo.com (K.M.); calin.vaida@mep.utcluj.ro (C.V.); zoltan.major@eeg-emg.ro (Z.M.); nicolae.plitea@mep.utcluj.ro (N.P.); giuseppe.carbone@unical.it (G.C.); 2 Municipal Clinical Hospital Cluj-Napoca, 400139 Cluj-Napoca, Romania; 3 DIMEG, University of Calabria, 87036 Cosenza, Italy

**Keywords:** robotic rehabilitation, stroke rehabilitation, risk assessment, safety assurance, robot design, fuzzy logic

## Abstract

A few decades ago, robotics started to be implemented in the medical field, especially in the rehabilitation of patients with different neurological diseases that have led to neuromuscular disorders. The main concern regarding medical robots is their safety assurance in the medical environment. The goal of this paper is to assess the risk of a medical robotic system for elbow and wrist rehabilitation in terms of robot and patient safety. The approached risk assessment follows the ISO12100:2010 risk management chart in order to determine, identify, estimate, and evaluate the possible risk that can occur during the use of the robotic system. The result of the risk assessment process is further analyzed using a fuzzy logic system in order to determine the safety degree conferred during the use of the robotic system. The innovative process concerning the risk assessment allows the achievement of a reliable medical robotic system both for the patient and the clinicians as well. The clinical trials performed on a group of 18 patients validated the functionality and the safe behavior of the robotic system.

## 1. Introduction

Stroke is usually defined as a sudden interruption of blood and nutrients flow to the brain. The longer time for which the flow is interrupted, the more severe are the consequences of the stroke [1]. Usual symptoms of an ongoing stroke are speaking difficulties, trouble in understanding, paralysis or in some cases numbness of arm or leg, difficulties in seeing with one or both eyes, headaches, or walking difficulties [2]. The survival rate after a stroke decreases with age and it is reported to be about 81.9% among men and 77.4% among women aged between 25 and 69 years old [3]. About 70% of stroke survivors suffer a certain level of upper limb disability [4] and every survivor needs a qualified person to deliver a specialized training in order to reduce or eliminate the disability. Post-stroke rehabilitation of upper limb is usually carried on through repetitive motions of the impaired limb in order to regain the capability to perform daily tasks using the disabled limb.

Given the growing lifespan of the population [5], the number of people suffering a stroke is expected to heavily increase in the near future, yielding a large number of qualified personal to work in post-stroke rehabilitation of survivors. In order to overcome this difficult situation, robotic structures for post-stroke rehabilitation of upper or lower limb started to be developed, being a suitable aid for the kinetotherapist performing the repetitive rehabilitation motions. In the last decades, a series of robotic structures for medical rehabilitation of the upper limb have been developed and analyzed by Huang [6], Al-Fahaan [7], Vaida [8,9], Carbone [10], Görgülü [11], Husty [12], Berceanu [13], Tarnita [14], Gherman [15], Tucan [16] and furthermore systematically reviewed by Ona [17,18], Baur [19], Onase [20], and Rehmat [21]. Some significant research prototypes are presented below.

Li et al. [22,23] propose a 5-degrees of freedom (DOFs) robotic structure (Co-Exoskeleton) composed of serial PPRRRP and RRP (where P stands for Prismatic joint and R stands for Revolute joint) mechanisms having five active revolute joints and four passive sliding joints distributed along the upper-body. The robotic structure is able to perform the adduction/abduction, flexion/extension and internal/external rotation of the shoulder, internal/external rotation of the forearm and flexion and extension of the elbow. The entire robotic structure is composed from an adjustable turntable, a cantilever, and the exoskeleton. The exoskeleton is mounted on a liftable column adjustable to different patients’ heights. The cantilever can rotate the exoskeleton to fit different shoulder types. The exoskeleton is suitable both for right arm and left arm rehabilitation. The advantage of this device is that it uses a binding vest to fix the patient to the chair and the modular design of the structure allows the use of the robotic device either as a shoulder rehabilitation device with 3DOf or as an elbow rehabilitation device with one DOF. The authors stated that improvements must be made to the kinematic model of the rehabilitation device in order for this to be implemented in the control system of the exoskeleton.

NESM [24] is an exoskeleton for shoulder and elbow rehabilitation mounted on a mobile platform. The robot contains four active revolute joints (three for the shoulder and one for the elbow). The motions performed with the exoskeleton are adduction/abduction/flexion/extension/internal rotation/external rotation of the shoulder and flexion/extension of the elbow. The robot has a modular structure divided into three sections: shoulder section, arm section and elbow section and it uses two main training control strategies: *robot-in-charge* (the robot performs preplanned motions with joint in position control) and *patient-in-charge* (the robot operates using torque mode control enabling the robot to adapt to spontaneous motions of the patient’s arm). The advantage of this rehabilitation system is that it benefits of a complex control system, able to adapt to various rehabilitation motions and to different anthropometric characteristics of the human arm. Future work regarding this device implies development of algorithms for inertia compensation. Also, the mass distribution in the mechanism is important when designing an exoskeleton (the mas of the moving structure is 12 kg) due to the fact that in the case of a power failure it is possible that the entire weight of the device to be projected onto the patient’s arm.

ETS-MARSE [25] is a redundant robot for the rehabilitation of the upper limb with 7-DOF. The design of the robotic structure is inspired from the human anatomy in order to offer a comfortable rehabilitation procedure for the patient. The shoulder joint is mimicked using three active revolute joints, the first one for adduction/abduction, the second one for flexion/extension and the third one for internal/external rotation of the shoulder. The elbow joint motion is performed using one active revolute joint. The other three DOFs are for pronation/supination of the forearm, radial /ulnar deviation and flexion/extension of the wrist. The control of the robotic system has the ability to adapt to different forces from the system through integration of an external force observer to identify the force of the patient. The advantage of this robotic system is the capability to perform every motion of the human arm due to its redundant configuration. At the time of research, the system was only tested as a passive rehabilitation device making it suitable only for the rehabilitation of the patients that recover some motricity after the stroke.

A medical rehabilitation system should be of a low degree of complexity both in design (low number of active mechanisms and actuated joints) and control (the number of possible failure modes increases with the degree of complexity of the control system). This paper presents a 4 DOF robotic system for elbow and wrist rehabilitation that uses an industrially tested and accepted control system able to overcome most of the risks associated with the rehabilitation task.

A considerable effort in ongoing research activities is spent on providing improved characteristics in terms of efficiency of manipulation and motion trajectories of the robotic structures used in upper limb rehabilitation. Along these technical characteristics of the robotic structures used as medical devices, some aspects regarding the social acceptance and the assured safety during the medical procedure need to be carefully analyzed and optimized (if necessary). Even though for a high safety degree, a complex sensor system should be used (use of internal sensor system to initialize the robotic system and monitor the behavior of the system; use of external observers to monitor the interactions between the robotic system and the patient; use of biosensors mounted on the patient to monitor the forces and displacements during the rehabilitation process) attention should be concentrated into creating a comfortable environment for the patient whom may be overstressed by prolonged preparation times before the rehabilitation procedure (time spent in mounting biosensors or electrodes on the impaired limb or use of brain computer interface that uses multiple electrodes) or the uncomfortable position during the procedure altering in the end the effects of the procedure.

Safety is one of the most important aspects of a medical device due to device’s working environment and conditions. Being a device that shares its workspace with the user (patient) a robotic system for upper limb rehabilitation should comply with every safety-related regulation (ISO13482 [26], ISO10218 [27], IEC 6061-4-44:2015 [28], ISO 12100:2010 [29], IEC 80601-2-78:2019 [30]), but sometimes this may not be sufficient to provide a safe robotic rehabilitation system both for patients and clinical personal operating it, for this, means of identifying, estimating and evaluating the risk should be used. One way to identify and overcome most of the risks implied by working with a medical device is to follow a risk assessment process. The output of a risk assessment is given by the severity of the hazards and the probability of the identified hazards (risks) and it should provide the means to achieve a safer device. Achieving safety through risk assessment is a method preferred by some researchers [31,32,33,34] because it provides statistical data without the need of a complex mathematical model to compute the results, based on the opinion of different persons from different research domains.

This paper presents a risk assessment process of a parallel robot for lower limb rehabilitation in order to identify different possible unsafe situations that can occur during the progress of the rehabilitation process. The risk assessment follows the risk management chart provided by ISO12100:20100 referring to safety in machinery. A fuzzy logic inference algorithm is used in order to avoid the uncertainties in human thinking in terms of providing objective information regarding the functionality of the robotic system. The ParReEx robotic system for wrist and elbow rehabilitation of stroke survivors is presented in terms of kinematic scheme and the experimental model. In the end, the safety provided during the rehabilitation procedure is validated by clinical trials using the robotic system.

The second section of the paper refers to Materials and Methods and it provides data regarding the robotic elbow and wrist rehabilitation system. Also a risk assessment process is described in order to identify the hazards that may occur during the rehabilitation process followed by the determination of the risk level for each hazard using fuzzy logic. The third section of the paper refers to the Results obtained during the research process and it provides data regarding the experimental model of the elbow and wrist rehabilitation robotic system and the functional validation of this robotic system by means of experimental tests performed in hospital environment on real patients. The fourth and fifth part of the paper contains the discussions and the conclusions regarding the research work presented within the paper. 

## 2. Materials and Methods 

### 2.1. ParReEx-Parallel Robotic Structure for Elbow and Wrist Rehabilitation

**Figure 1:**
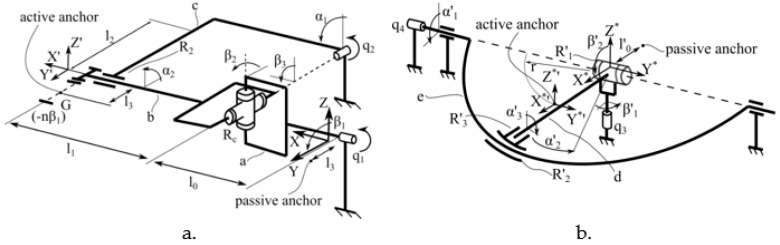
Kinematic scheme of the ParReEx robotic structure ((**a**) kinematic scheme of the elbow rehabilitation module, (**b**) kinematic scheme of the wrist rehabilitation module) [37].

ParReEx [35,36] robotic system is composed from two independent parallel modules: ParReEx-elbow (Figure 1a) for rehabilitation of the elbow and ParReEx wrist (Figure 1b) for rehabilitation of the wrist.

The module for elbow rehabilitation has 2 DOFs and is composed of two kinematic chains. First kinematic chain is of type RU (where R stands for Revolute joint and U stands for Universal joint) and has its origin in the fixed coordinates system XYZ (Figure 1a). The active joint of this kinematic chain is q_1_, a revolute joint collinear with OX axis. Using link a the motion of q_1_ is transmitted to the passive cardan joint R_c_ which is connected with the mobile coordinates system X’Y’Z’ through link b. The second kinematic chain is of *RR* type and is actuated by q_2_ revolute joint connected to link b through link c and passive revolute joint R_2_. The module for elbow rehabilitation performs the pronation and supination rehabilitation motion of the forearm using q_1_ joint and flexion and extension motion of the forearm using q_2_ joint. 

The module for wrist rehabilitation is a 2 DOFs mechanism too. The fixed coordinate system is placed at the intersection of the rotation axes of active revolute joint q_3_ and passive revolute joint R’_1_ and the mobile coordinate system is placed at distance *r* (Figure 1b). The center of rotation for the wrist is placed in the fixed coordinates system. The mechanism is also composed of two kinematic chains, first one is of RR type actuated by revolute joint q_3_ connected to the mobile coordinates system through passive revolute joint R’_1_ and link d. The second kinematic chain is actuated by q_4_ revolute joint that rotates the circular guide *e* around the O*Y* axis. The intersection of d link with the circular guide *e* is made through two revolute joints, R’_2_ (which slides on the circular guide e) and R’_3_ (passive revolute joint around O*’X*’ axis). The flexion and extension of the wrist is obtained through the kinematic chain actuated by q_3_ and the abduction and adduction through the kinematic chain actuated by q_4_.

### 2.2. Identification of Risks of the Rehabilitation Process Using ParReEx Structure

**Figure 2:**
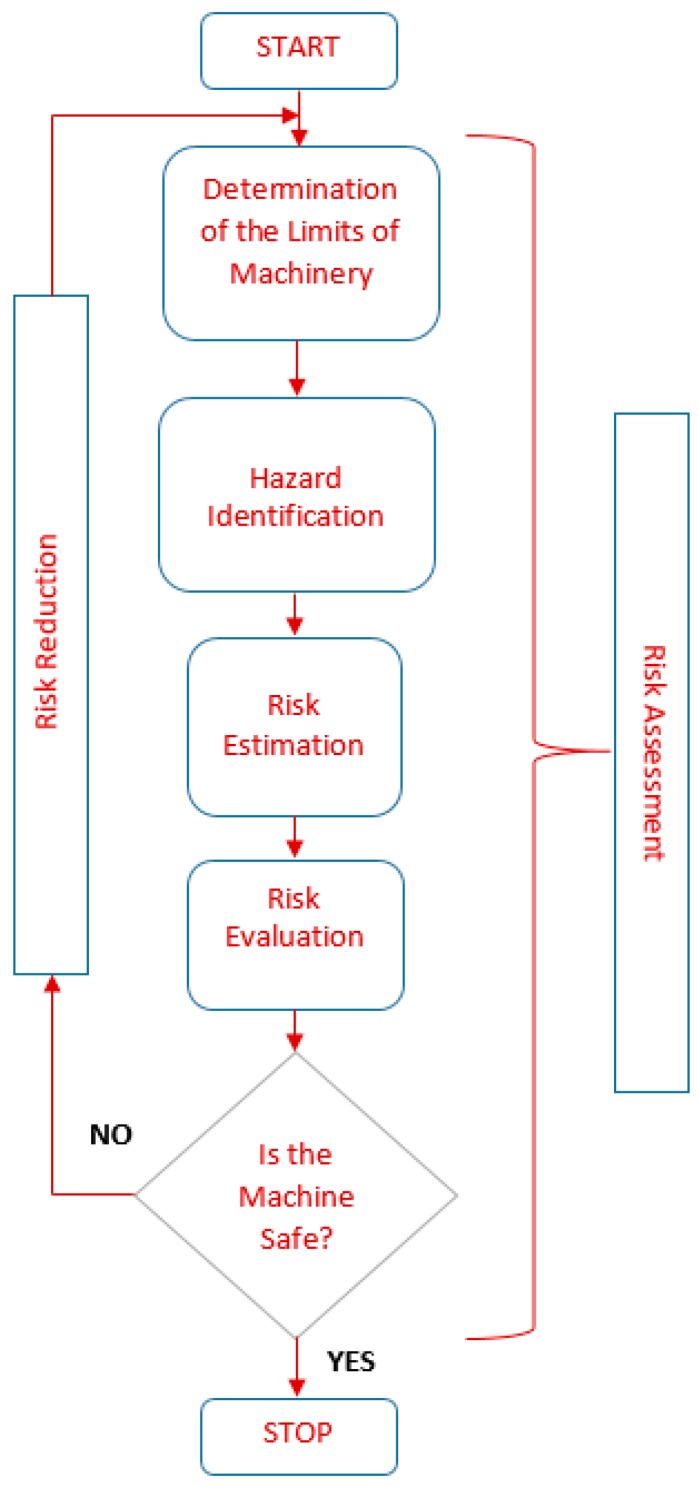
Risk management flow chart regarding the risk assessment process according to ISO12100:2010.

Risk assessment is an important step in risk management and it has the scope of determining risk context and acceptability, usually by comparison with a similar risk [38]. ISO12100:2010 [29] referring to “Safety of Machineries, General principles for design, Risk assessment and risk reduction” delivers a series of terms, useful in analyzing the degree of safety during the design of the machinery. The flow chart of a risk management analysis is given in Figure 2.

#### 2.2.1. Determination of the Limits of the Machinery

First step in the risk assessment process is to define the limits of the machinery for this level of interaction between the patient and the robot should be defined.

Ogorodnikova [39] defines in Table 1 the interaction levels between the robot and the human operator.

Depending on the position in Table 1, a robotic device should fulfil different safety requirements. During the rehabilitation procedure, the patient shares the same workspace with the robot, resulting in a collaboration between the rehabilitation system and the patient. When the robotic system runs in normal conditions, the patient is seated near the rehabilitation system using either a liftable chair or a wheelchair, depending on the state of the patient, with the right arm attached either to the wrist rehabilitation system or to the elbow rehabilitation system. At the debut of the rehabilitation procedure, the patient has none or little control of the impaired limb, so the degree of help coming from the robotic rehabilitation system should be accordingly dosed. As the patient regains some capability to perform the motions by itself the degree of help coming from the robot would be gradually reduced.

**Figure 3:**
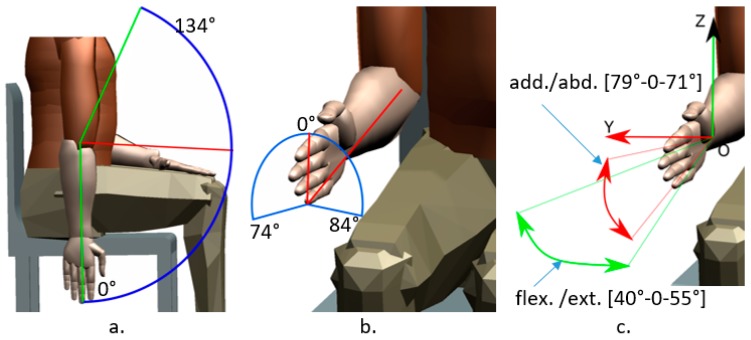
Motion ranges for the elbow rehabilitation module ((**a**) flexion/extension, (**b**) pronation/supination) and for wrist rehabilitation device ((**c**) flexion/extension/adduction/abduction).

The limits of the robotic structure are given by the maximum range (degrees—[°]) of each rehabilitation motion, the range of motion performed using ParReEx are given in Table 2 and illustrated in Figure 3.

The design of the robotic structure is based on the anthropomorphic data collected from systematical reviews and clinical studies regarding the stroke survivors that received medical care regarding the rehabilitation of the elbow and wrist [40].

#### 2.2.2. Hazard Identification 

After the limits of the machinery have been defined, according to the flow chart from Figure 3, an analysis regarding the hazards must be carried on. Every hazard that can occur must be identified or else will be missed in the risk reduction stage. In the following lines are given the types of hazards that have been analyzed.

Mechanical hazards

- M1: The arm of the patient may be crushed by the moving mechanism. This hazard can occur due to incorrect posture of the patient, wrong parameters introduced in the control system (not suitable for patient movement capabilities), it can even be a consequence of power failure.
- M2: The patent may bruise or cut its hand in the sharp edges of the components of the robotic structure. This may occur due to improper design of mechanical components of the robotic system.
- M3: Motion limit exceeded. The robot either exceeds the moving capabilities of the patient or the moving mechanism goes over the end stroke limiters.
- M4: The patient may crush into the robotic structure. Usually, the patient is either carried using a wheelchair or if he can walk may suffer sudden loss of equilibrium.
- M5: The patient may have sudden spasms resulting in incontrollable motion of the body (e.g., Parkinson disease). The robot should be able to overcome and resist to this situation, allowing in the same time easy removal of the patient’s arm from the rehabilitation device.

Electrical hazards

- E1: Risk of electrocution of the patient.
- E2: Harming the patient due to sensor malfunction.
- E3: Crush the arm of the patient caused by the malfunction of stroke limiters.
- E4: Risk of short circuit.
- E5: Overloading the rehabilitation device.

Thermal hazards

- T1: The patient may suffer burns by coming into contact with overheating parts of the robot.

Noise hazards

- N1: Due to the fact that the robot works quite close to the patient, the sound created by moving mechanisms may create some discomfort for the patient.

Vibration hazards

- V1: Patient may be harmed by loose parts from the robotic system.
- V2: Patient may be harmed by uncontrolled vibrations of the mechanism.

Ergonomic hazards

- ER1: The patient may fall due to the fact that he is in a wheelchair.

#### 2.2.3. Risk Estimation

After the hazards have been identified, it is time to estimate the probability and the severity of each risk, in order to do this, severity and probability categories need to be defined. In Table 3 the probability categories are defined and Table 4 contains the categories of severity.

**Figure 4:**
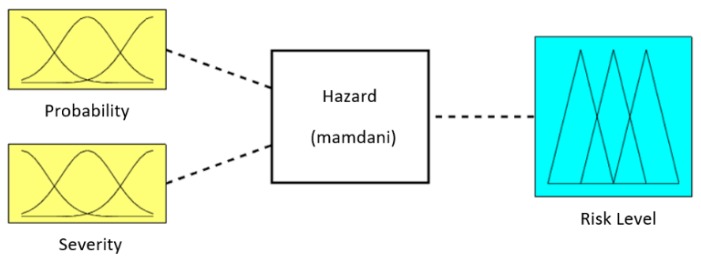
The structure of Mamdani FIS (Fuzzy Inference System) provided by MATLAB.

**Figure 5:**
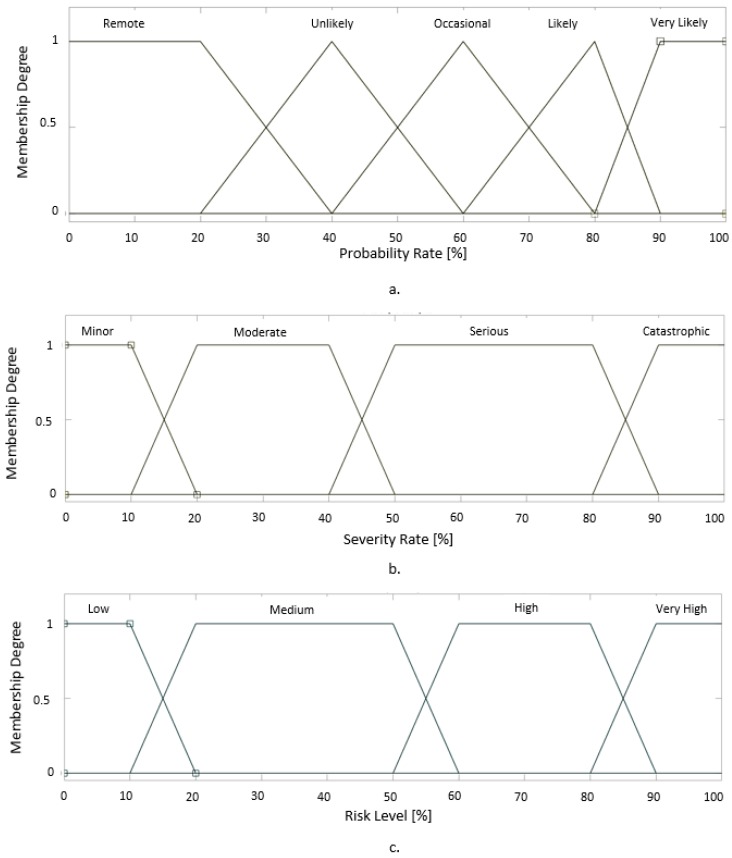
The membership function of the Fuzzy Inference System (FIS) ((**a**) Input membership function for Probability of the hazard, (**b**) Input membership function for the severity of the hazard, (**c**) Output membership function for the Risk Level of the hazard).

In order to determine the risk level of each identified hazard, input membership functions have been designed for Probability and Severity using Mamdani FIS (Fuzzy Inference System) provided by MATLAB [41] (Figure 4). The membership function for Probability is given in Figure 5a and for Severity in Figure 5b.

The membership functions for Probability were modeled using triangular functions. For the membership function of probability rate Equation (1) was used, where the numerical values are taken from Table 3.

(1) $$\mu \left(x\right)=\left\{\begin{array}{ll} 0, & x\leq a\\ \frac{x-a}{m-a}, & a<x\leq m\\ \frac{b-x}{b-m}, & m<x<b\\ 0, & x\geq b \end{array}\right\}$$

where, *a* represents the lower limit of the interval, *m* represents the core of the membership (the core of the membership is the point where the degree of the membership function is maximum while all the others membership functions are 0, best example can be seen in the membership function of “Likely” that differs from the other membership functions, due to the fact that the core has to be positioned when the other membership functions are 0, the asymmetry is caused by the fact that the other functions are defined on the same interval length while the interval of membership function of “Likely” has a smaller length), *b* represents the superior limit of the interval within which the membership functions degree is different from 0.

The membership functions for Severity and Risk Level were modeled using trapezoidal functions. For the membership function of probability rate Equation (2) was used, where the numerical values are taken from Table 4.

(2) $$\mu \left(x\right)=\left\{\begin{array}{ll} 0, & \left(x<a\right)or\;(x>d)\\ \frac{x-a}{b-a}, & a\leq x\leq b\\ 1, & b\leq x\leq c\\ \frac{d-x}{d-c}, & \;c\leq x\leq d \end{array}\right\}$$

where, *a* represents the lower limit of the interval, *b* represents the inferior core value of the membership, *c* represents the superior core value of the membership and *d* represents the superior limit of the interval within which the membership functions degree is different from 0.

The output membership function of “Risk Level” is defined in Figure 5c using the same scale as the previous membership functions. 

In order to obtain the risk level value, every degree of “Probability “is connected using logical operator “AND” with every degree of “Severity” as described in Table 5.

**Figure 6:**
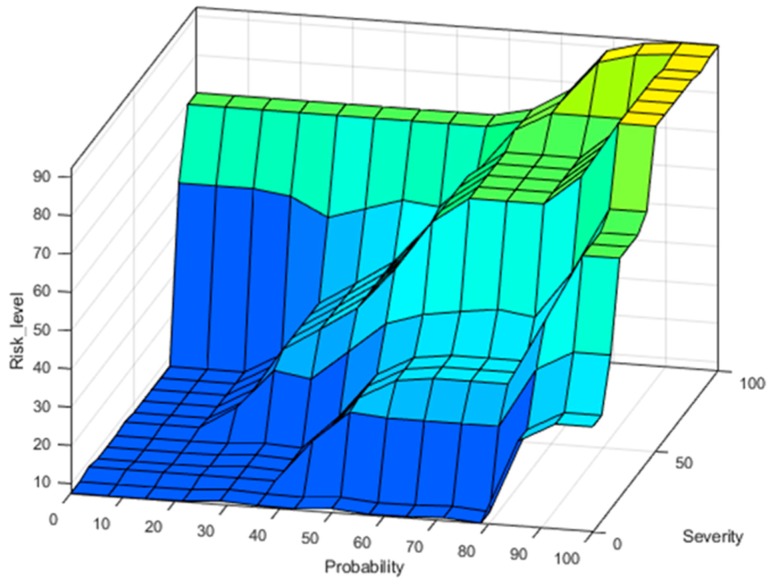
Risk level surface with respect to Probability and Severity membership functions.

Using the above-mentioned membership functions for inputs and outputs, correlated with the FIS rules form Table 5, the surface distribution of Risk Level is represented graphically in Figure 6.

#### 2.2.4. Risk Evaluation

To evaluate the risk of every event, each hazard identified above needs to be the subject of a FIS. For this, using expertise of 15 different researchers from mechanical (7 researchers), electrical (3 researchers) and medical domain (5 clinicians) [16] some mean values in terms of severity and probability have been obtained for each hazard. The values were obtained using the questionnaire provided in Appendix A. Table A1 was used to record the probability and Table A2 was used to record the severity of each hazard. The final values obtained averaging the questionnaire data are given in Table 6.

**Figure 7:**
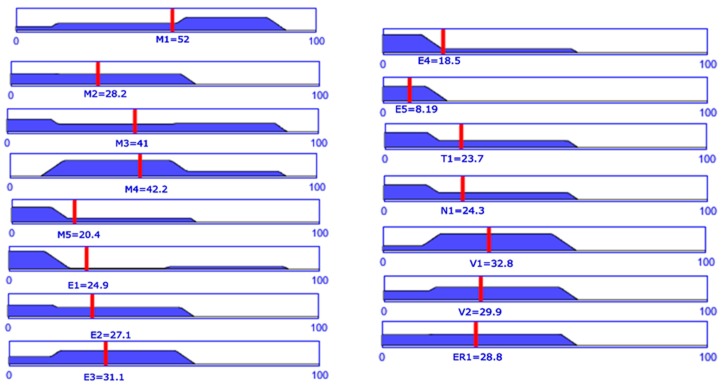
Risk level for each hazard computed using data from Table 7.

The Risk Level degree is computed by using mean value for each hazard as input in the FIS and following the defined rules. The result for each hazard is represented in Figure 7.

#### 2.2.5. Risk Reduction

After the risk assessment process has been concluded, a series of hazards that present medium or high risk resulted. In order to build a safe robotic system, the development team decided to find means to reduce every identified hazard. To reduce risk of M1, some proximity sensors should be mounted in the areas where collisions may occur, each axis should be equipped with torque sensors, the range of each motion should be limited by controller and an external measuring system should be used to directly measure the motion of the patient’s limb. To reduce the risk of M2, soft materials should be used to cover the parts that may be coming in the vicinity of the patient and again proximity sensors should be mounted in the areas where collisions may occur. To reduce M3 torque sensors should be mounted on each axis, the motion of each axis should be constrained mechanically and from the control unit. To reduce M4 end-stroke sensors should be mounted on each axis, and proximity sensors should be mounted in collisions susceptible areas. To reduce M5 the robotic system should be equipped with a passive mode switch, which allows the clinician to easily remove the patient’s arm from the robotic structure. To reduce E1, E4 and E5 proper regulated protection for the electric components of the system should be used. To reduce E2 and E3 a secondary sensor system should be used to check the position between the robot and the patient. T1 may be reduced by avoiding the use of parts that can generate or store heat when in use or avoiding using this kind of parts in areas where the patient in is direct contact with the robotic structure. V1 and V2 may be reduced by instructing the users to carefully check the robotic system for improperly fixed components before every procedure, and ERH1 may be reduced by using of harnesses to keep the patient in position during the rehabilitation process.

## 3. Results

In order to assure the safety of the ParReEx robotic structure the above-described method was used. After some means of reducing the risk have been provided the development of the experimental model of the robotic structure started. The safety features defined above are implemented in the developed experimental model of the ParReEx structure presented in the following paragraphs along with the experimental functional validation of the robotic structure.

### 3.1. The Experimental Model of the Parreex Robotic System for Wrist and Elbow Rehabilitation

**Figure 8:**
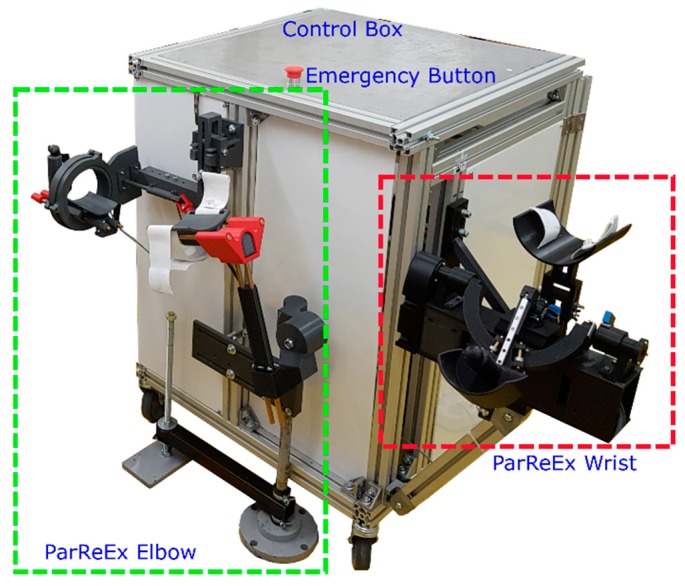
The experimental model of ParReEx robotic system.

The ParReEx rehabilitation system is composed of two modules (Figure 8), one for elbow rehabilitation and the other one for wrist rehabilitation. Both modules share the same control box and the same control interface. The components of the robotic structure are mostly composed of 3D printed parts, providing in the same time, low fabrication times, high shape complexity which ensures a minimum number of moving components, low resilience material designed to break in case of emergency and the superior material softness to the parts manufactured from metal. Plastic was used mostly in the areas where the patient is in intimate contact with the robot (see anchor points), but for transmitting motion from the motors to the rehabilitation mechanism also some metal parts were used (brass bars, screws, gears). The mechanisms of the rehabilitation structure are actuated using 4 motors, controlled using a PLC and 2 drivers provided by Berneker & Reiner [42]. The control unit is integrated into a control box that acts in the same time as a support base for the entire robotic structure. In the case of unpredicted situations or emergencies the box is equipped with an emergency button placed on top of the box (see Figure 8).

**Figure 9:**
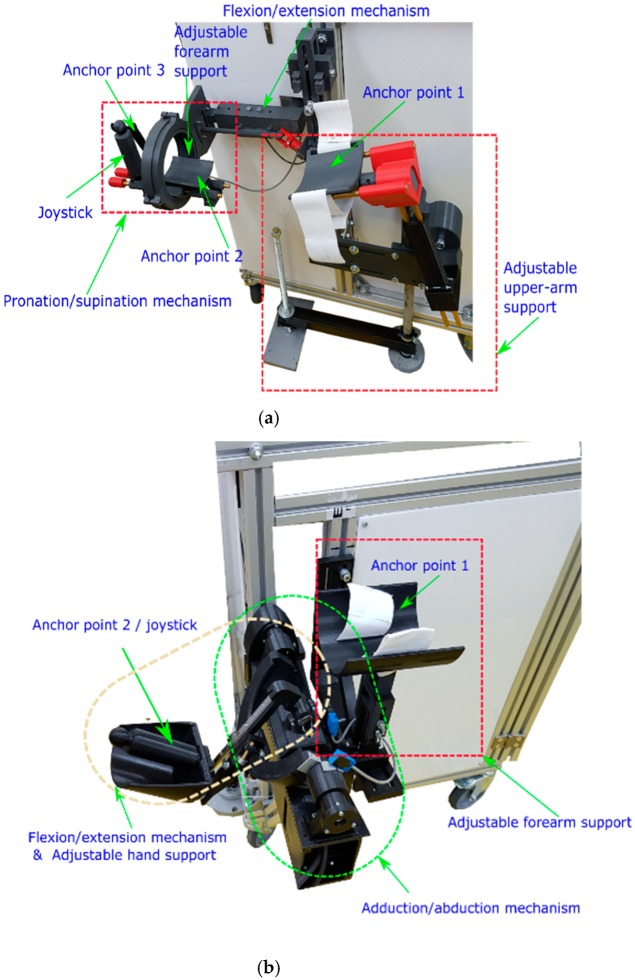
The experimental model of ParReEx robotic system ((**a**) elbow rehabilitation module, (**b**) wrist rehabilitation module).

The module for elbow rehabilitation uses 3 anchor points to secure the patient’s arm during the rehabilitation procedure. One of these points is placed on the upper arm, one on the forearm and the third one on the hand of the patient (Figure 9a). The patient is carefully positioned in a sitting position in the vicinity of the robotic system (using liftable seat or wheelchair depending on the degree of severity of the impairment), and the right arm of the patient is attached to the rehabilitation device, using the previously described anchor points. The arm of the patient is kept secure in the correct position using Velcro elastic bands. The patient must grip the joystick of the elbow rehabilitation module in order to ensure the correct position of the arm during the elbow rehabilitation procedure. The motions for the elbow rehabilitation module are flexion/extension/pronation and supination. The flexion and extension is performed using the flexion/extension mechanism actuated through a conical reduction box by a servomotor. The pronation and supination motion is performed using also a servomotor but this time the motion is transmitted to the mechanism using a U joint and a spur gearbox.

The module for wrist rehabilitation has two anchor points, one placed on the forearm and the other one on the hand (Figure 9b). To keep the forearm in the correct position a Velcro elastic band is also used, and the patient must grasp the joystick of the wrist rehabilitation module using the right hand. The motions for the wrist rehabilitation module are flexion/extension/adduction and abduction. The flexion and extension is performed using the flexion/extension mechanism actuated by a servomotor. The abduction and adduction motion is performed using a second servomotor but this time the link in motion is a circular guide that makes a sliding joint with the flexion/extension mechanism.

**Figure 10:**
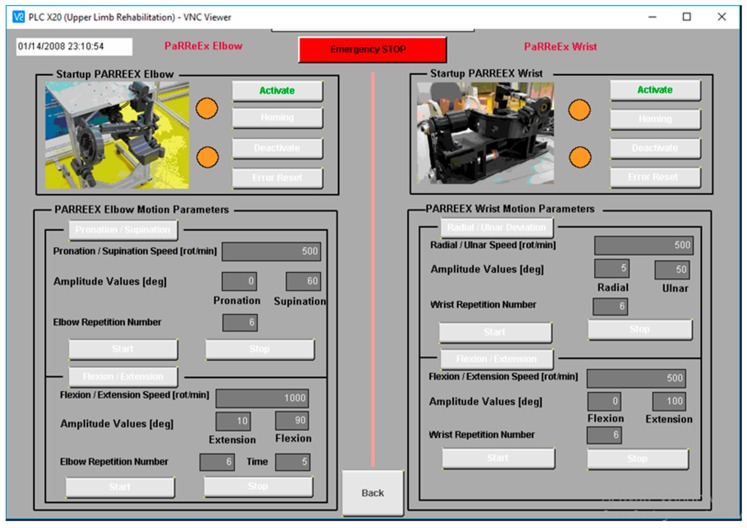
The Graphical User Interface (GUI) of ParReEx designed using Automation Studio [36].

The robotic system is controlled using a Graphical User Interface (GUI) designed to help the clinicians during the use of the rehabilitation system (Figure 10). The GUI is divided into two sections one for controlling the ParReEx-Elbow module and the other one for controlling the ParReEx-Wrist module. The state of the robot can be visualized using the 4 LED’s on the interface (Orange—the motors are not initialized, Red—the motors are in error state, Green—the motors are initialized and ready to receive commands, Blue—the motors are in motion). To activate each of the rehabilitation modules, the “Activate” button must be pressed, enabling the command that activates the motors and releases the brake. In order to initialize the robotic structure “Homing” button must be pressed and each axis will perform a motion towards the sensor position until the signal from the axis initialization sensor is received. After the axes are initialized the robotic system is ready to perform the rehabilitation task, for this, the range of motion for the patient’s upper limb should be provided in the GUI in terms of amplitudes, speed and number of repetition. After the parameters have been provided a dry run is suggested to check the behavior of the rehabilitation module. After the rehabilitation motion is correctly executed the patient may be seated with the arm placed in the rehabilitation module. In case of emergency, the GUI provides the possibility to cut the power of the controller by pressing the “Emergency Button”. In this case, the patient should be removed from the rehabilitation robot and the system must be re-initialized. If during the functioning of the robotic system, an error occurs, this can be acknowledged by pressing the “Error Reset” button. At the end of the procedure, if no other procedures are performed the system may be turned off by pressing the “Deactivate” button and turning off the main switch placed inside the control box.

### 3.2. Functional Validation of Parreex

For validating the functionality of ParReEx robotic system, tests involving human patients have been performed. The approval for performing these tests was given by Institutional Regulatory Board (IRB) of Municipal Cluj-Napoca Hospital, Romania in 1 August 2019. Before performing clinical tests in the hospital environment, the robotic system was previously analyzed and approved for hospital use by the clinicians and for functional validation purposes, the clinicians selected a number of 18 patients with different pathologies and experiencing various degrees of disability of the right upper limb. Data regarding the patients involved in the clinical trials are given in Table 7.

The experimental setup consisted of the rehabilitation system and a wheelchair to move the patients between the wrist and elbow rehabilitation modules, a human operator for the robotic structure, a kinetotherapist to evaluate the patient and define the rehabilitation plan and (when necessary) a stretcher-bearer to help in moving the patient from one rehabilitation module to another. The experimental tests were conducted for each patient on a 7 days period of time during which time the patient was admitted in the hospital. Table 8 provides data regarding the mean, minimum and maximum values recorded during the clinical trials, the following notions were used:

- No. of series—the number series of repetitions for each rehabilitation module;
- Rep/series—the number of repetitions for each series;
- Wrist F—flexion amplitude of the wrist [°];
- Wrist E—extension amplitude of the wrist [°];
- Wrist Add.—adduction amplitude for the wrist [°];
- Wrist Abd.—abduction amplitude for the wrist [°];
- Elbow F—flexion amplitude for the elbow [°];
- Elbow E—extension amplitude for the elbow [°];
- Elbow P—pronation amplitude for the forearm [°];
- Elbow S—supination amplitude for the forearm [°].

The angular amplitudes given in the following table were actually the input data for the robotic system, introduced in the specially designed sections in the GUI.

**Figure 11:**
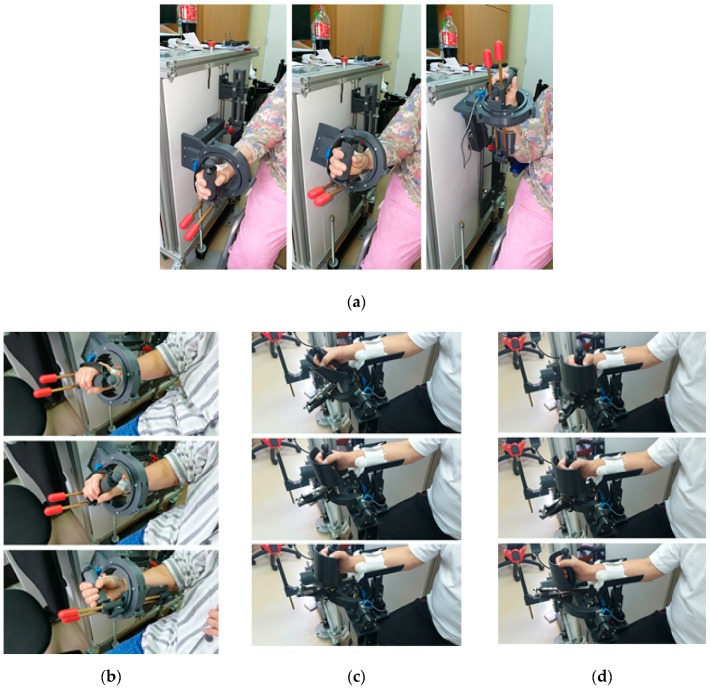
Snapshots from the experimental tests using ParReEx ((**a**) ParReEx-elbow flexion/extension, (**b**) ParReEx-elbow pronation/supination, (**c**) ParReEx-wrist adduction/abduction, (**d**) ParReEx-wrist flexion /extension).

The tests performed using the PArReEx robotic system in the hospital environment, proved the functionality of the robotic system and the capability of performing rehabilitation motions of the elbow and wrist following a rehabilitation chart provided by a kinetotherapist. The plan imposed by the kinetotherapist implied a gradual increase in the amplitude of the performed motions. Snapshots taken from the videos recorded during the testing phase can be seen in Figure 11.

The safe behavior of the robotic system was proved through “zero events” during the experimental tests (no scratches on the patient, no concussions caused by crashing into the robotic structure, no bruises, no burns, no noise discomfort for the patient and the personal, no electrical shocks or discharges and most important no discomfort for the patient). The robotic system was largely accepted both by the patients and by the clinicians.

## 4. Discussion

In the existing regulatory documentation are not clearly specified ways of assuring safe behavior of robotic systems used as rehabilitation devices. Thus, some of the regulation provide a series of paths to follow in order to be able to provide a solution reliable enough to successfully perform the given rehabilitation task, but for this, expertise from different research domains, such as mechanical, electrical, and medical, is required. 

This paper presents a method of achieving a safer behavior of the robotic structure used in rehabilitation of the wrist and elbow by means of risk assessment and fuzzy logic inference system. First, a risk assessment process is carried on in order to identify the possible hazards that can occur when using a wrist and elbow rehabilitation robotic system. After the hazards were identified, a FIS is used in order to estimate the risk of each hazard. For this, membership functions regarding the inputs (Severity and Probability of the hazard) and output (Risk level) of the FIS are defined using triangle and trapezoidal variance. After the inputs and outputs of the system have been defined, the inference rules of the FIS are defined with respect to a risk assessment matrix, and the output of the FIS is obtained and graphically represented as a surface graph. In order to estimate the risk level of every identified hazard, expertise of 15 mechanical, electrical, and medical experts is used to determine the mean score regarding the Severity and Probability of each hazard. The obtained mean score is used afterwards as numerical inputs for the FIS in order to determine the risk level for each hazard. The obtained risk level for each identified hazard was graphically represented and ways in reducing the overall risk of every hazard were provided.

With respect to the safety requirements given in the risk reduction process, the experimental model of the robotic system ParReEx was presented in terms of mechanical structure, control system and GUI.

In the development of the experimental model the following measures were taken to overcome the above-identified risks:

- Risk M1, M3, and M4: inductive proximity sensors have been mounted in order to constrain the mechanism within the allowable limits, no torque sensors were used but instead the drivers of the motors provided information regarding the forces in the mechanism.
- Risk M2: the anchor points were manufactured from plastic (3D printed) and covered with soft materials (sponges and cotton), inductive sensors have been mounted in order to constrain the mechanism within the allowable limits.
- Risk M5: when the Emergency button is pressed the robotic system allows easily removal of the patient from the device (the mechanism remains in the position).
- Risks E1, E4 and E5: properly 220 V encapsulated power supply was used, the power supply for the motors is 80 V and properly encapsulated cables have been used to supply the motors. The large case box containing the control system was used as grounding.
- Risk E2 and E3: the room where the robotic system was installed did not permit the use of external sensor systems during the patient experimental runs, but the research team achieved two external sensor systems one of them using cameras and the other one using goniometers.
- Risk T1: was reduced by use of plastic materials in the patient-robot contact areas.
- Risk V1 and V2: proper instruction manual was provided.
- Risk ERH1—no harnesses were necessary, instead some pillows were used to adjust the patient position during the procedure.

Finally, in order to validate the functionality of the robotic structure the ethical approval for performing test using humans was requested and obtained. Experimental data was collected from 18 patients each of them hospitalized for a time period of 7 days. The clinical profile of each patient was provided as a table.

The main objective of the in hospital tests was to validate the functionality of the robotic system for elbow and wrist rehabilitation, system previously tested on healthy human subjects in the laboratory environment. Most important aspect of using the robotic system in the hospital environment was the feedback obtained from the patients and the clinicians.

The patients were given a consent form prior to the robotic-assisted therapy, so all had to accept voluntarily this type of treatment, but the feedback was better than initially expected. Everyone showed a large interest and excitement in using a device never used before in the hospital. On the medical side, the overall time of each session was around 25 min which seemed to have greater benefits to the patient (as the exercises were performed with lower speeds) as compared to classical sessions which are spread on a period of only 5–8 min. On the motivational side, we noticed that patients exchanged opinions encouraging each other which seemed to help the entire group. The market of rehabilitation devices for upper limb rehabilitation is in continuous development and expansion, but there are not many devices undergoing clinical trials. Basteris [43] provides a list of 38 upper limb rehabilitation devices that were used in clinical trials using real patients, the number of the patients included in the clinical trials varying between 5 and 526. Colombo [44] provides data regarding clinical trials performed on a number of 12 patients accumulating 20 h of training with the robot, the clinical trials were performed on chronic patients. Abdullah [45] reports clinical trials performed on a number of eight patients accumulating 21.4 h of training with the robot but this time the patients were in acute phase. Stein [46] reports clinical trials involving 12 chronic phase patients achieving 18 h of training with the robot. Chang [47] report clinical trials on 20 chronic patients reveling also 18 h of training with the robot. The experimental tests performed with ParReEx robotic system included 18 acute and chronic patients and accumulated 1890 min (31.5 h) of training with the elbow rehabilitation module and 1260 min (21 h) of training with the wrist rehabilitation module. Prior to the clinical trials, the accuracy of the robotic system was tested using runs with healthy human subjects. The experimental setup necessary for determining the accuracy of the system was composed of the robotic system and an external monitoring system provided by Biometrics [48] a sensor system composed from a series of goniometers able to read the angular amplitudes during the rehabilitation process. A series of dry runs (without the human subject) were performed in order to obtain the mechanism accuracy without external forces. The obtained accuracy was obtained between 0.5° and 1° for elbow rehabilitation module and 0.5° and 1.5° for the wrist rehabilitation module. The positioning error increased when attaching the human subject in the rehabilitation device until 3°, due to the elasticity of some of the components parts of the device but this error was considered as a characteristic to improve in the future and has been taken into consideration during the clinical trials. During the clinical trials, the functionality of the robotic system was proven due to no major issues encountered, even though there were cases when the patient suffered muscular spasm due to Parkinson altering normal running conditions, but in most of the cases, the drivers of the motors interrupted the motion due to excessive torque detected. The advantage of performing rehabilitation using a modular robotic device that does not uses the patient’s arm as support is that in cases of malfunction or power failure, the mechanism stops and is kept in place by the gearboxes and the patient’s arm can be easily removed from the device. The robotic system was previously tested using healthy human subjects to intentionally apply excessive forces in the mechanism in order to block it and to test the possibility of removing the arm from the structure. Some irregularities were detected when the elbow joint was at 90° and to overcome this situation, easy removal of the arm anchor point (anchor point 1 in Figure 9a) was provided.

## 5. Conclusions

The overall behavior of the robotic system during the clinical trials was generally accepted by the clinicians, patients, and operator. The improvement in the patient neurological disorder is yet to be analyzed by the clinicians in order to validate the robotic system from medical point of view as a viable rehabilitation solution for post-stroke patients (or other neurological diseases that cause limb impairment), but the authors consider that the experimental model of ParReEx passed the functionality test. During the clinical trials, some aspects that may be improved were recorded. Future work will be focused on improving the control system, the GUI, and improving the quality of some components of the experimental model of ParReEx, such as material used in anchor points, the Velcro bands should be covered with a softer material, the wheelchair works fine for the chronic phase patients, but in the case of acute patients some extra fixtures should be considered. As stated before, a big preconized problem was the acceptance of the robotic system by the patients, but this proved to be a minor inconvenience and only in the first phase of trials, the research team considers that the general acceptance of the robotic system is due to the fact that the number of sensors has been reduced to minimum and no sensor was mounted on the patient during the procedure in order to reduce the preparation times (actually, patients didn’t need any preparation times) and to create a comfortable environment for the patient.

## Figures and Tables

**Table 1:** Robot-Human interaction levels [39].

Description	Human Role
Inside the operational robot workspace	Collaboration/Feeding
Outside operational zone, but inside restricted work zone	Teaching/Measuring
Inside or outside of the restricted robot work zone	Verification/Operation
Outside robot maximum reach	Monitoring/Observing

**Table 2:** Motion ranges for the elbow and wrist rehabilitation.

Targeted Area	Flexion [°]	Extension [°]	Abduction [°]	Adduction [°]	Pronation [°]	Supination [°]
Elbow	134	0	N.a	N.a	74	84
Wrist	40	55	71	79	N.a	N.a

°—degrees.

**Table 3:** The probability categories for the hazard occurrence.

Probability Rate	Explanation	Density
Very likely	Occurs often	More than 80
Likely	Occurs several times	60–90
Occasional	Will occur	40–80
Unlikely	May occur	20–60
Remote	May assume it will not occur	Less than 40

**Table 4:** The severity categories for the hazard occurrence.

Severity Rate	Explanation	Density
Catastrophic	Complete failure, death, loss of system	80–100
Serious	Severe injury, major system damage	40–90
Moderate	Minor injury, minor system damage	10–50
Minor	Less than minor injury, minor system damage	0–20

**Table 5:** FIS rules defined according to the risk assessment matrix.

Rule No.		Probability Is		Severity Is		Risk Level Is
1	**IF**	Very Likely	**AND**	Catastrophic	**THEN**	Very High
2		Likely		Catastrophic		Very High
3		Occasional		Catastrophic		High
4		Unlikely		Catastrophic		High
5		Remote		Catastrophic		Medium
6		Very Likely		Serious		Very High
7		Likely		Serious		High
8		Occasional		Serious		High
9		Unlikely		Serious		Medium
10		Remote		Serious		Low
11		Very Likely		Moderate		High
12		Likely		Moderate		Medium
13		Occasional		Moderate		Medium
14		Unlikely		Moderate		Low
15		Remote		Moderate		Low
16		Very Likely		Minor		Medium
17		Likely		Minor		Low
18		Occasional		Minor		Low
19		Unlikely		Minor		Low
20		Remote		minor		Low

**Table 6:** Mean values for Severity and Probability [16].

Hazard	Probability [%]	Severity [%]
M1	36	86.5
M2	29.7	48.5
M3	27.3	84
M4	45	53.5
M5	34.3	42
E1	21	81.3
E2	28.7	49.4
E3	33	48
E4	23.5	61.5
E5	33.3	31
T1	26.2	74.9
N1	46.6	26.5
V1	35.5	73.5
V2	31.5	65.5
ER1	30.3	72.5

**Table 7:** Data regarding the patients involved in the functional validation of the robotic system.

Patient No.	Gender	Age [years]	Hospitalization Period [days]	Neurological Disorder	Time from Diagnostic [months]	Time on Robot Daily [minutes]	
						*P*. elbow	*P*. wrist
1	male	72	7	Stroke	2	15	10
2	male	68	7	Stroke	9	15	10
3	female	70	7	Parkinson	24	15	10
4	male	73	7	Parkinson	24	15	10
5	male	67	7	Parkinson	6	15	10
6	female	75	7	Stroke	12	15	10
7	female	72	7	Stroke	6	15	10
8	male	76	7	Parkinson	30	15	10
9	male	71	7	Stroke	6	15	10
10	female	76	7	Parkinson	36	15	10
11	female	69	7	Parkinson	12	15	10
12	female	66	7	Parkinson	12	15	10
13	female	75	7	Brain surgery	1	15	10
14	male	67	7	Stroke	6	15	10
15	female	55	7	Parkinson	18	15	10
16	male	71	7	Stroke	12	15	10
17	female	70	7	Gouty arthritis	20 years	15	10
18	female	73	7	Stroke	16	15	10
Total functioning (in rehabilitation) time						1890	1260

**Table 8:** Mean, minimum, and maximum angular amplitudes recorded during the clinical trials.

Rehabilitation Description	Mean Amplitude	Min Amplitude	Max Amplitude
No of series	6		
Rep/series	10		
Wrist F [°]	3	0	20
Wrist E [°]	99	70	110
Wrist Add. [°]	7	1	10
Wrist Abd. [°]	45	20	50
Elbow F [°]	3	0	30
Elbow E [°]	97	70	110
Elbow P [°]	66	30	80
Elbow S [°]	70	40	80

## Appendix A. Questionnaire [Hazard Probability and Severity]

**Table A1:** How often would you consider the occurrence probability of the following hazards? (Please provide a numeric value in the interval provided).

Probability of a Hazard for the Wrist and Elbow Rehabilitation System		Please Provide a Value between 0 % of Happening and 100 % of Happening
M1	The arm of the patient may be crushed by the moving mechanism. This hazard may be created by incorrect posture of the patient, wrong parameters introduced in the control system (not suitable for patient movement capabilities), it can even be a consequences of a power failure	
M2	The patent may bruise or cut its hand in the edges of the components of the robotic structure. This may occur due to improper design of mechanical components of the robotic system.	
M3	Motion limit exceeded. The robot either exceeds the moving capabilities of the patient or the moving mechanism goes over the end stroke limiters.	
M4	The patient may crush into the robotic structure. Usually the patient is either carried using a wheelchair or if he can walk is often suspected by sudden loss of equilibrium.	
M5	The patient may have sudden spasms resulting in incontrollable motion of the body. The robot should be able to overcome and resist to this situation, allowing in the same time easy removal of the patient’s arm from the rehabilitation device.	
E1	Risk of electrocution of the patient.	
E2	Harming the patient due to sensor malfunction.	
E3	Crush the arm of the patient caused by the malfunction of stroke limiters.	
E4	Risk of short-circuit	
E5	Overloading the rehabilitation device	
T1	The patient may suffer burns by coming into contact with overheating parts of the robot.	
N1	Due to the fact that the robot works quite close to the patient, the sound created by moving mechanisms may create some discomfort for the patient.	
V1	Patient may be harmed by loose parts from the robotic system.	
V2	Patient may be harmed by uncontrolled vibrations of the mechanism.	
ER1	The patient may fall due to the fact that he is in a wheelchair.	

**Table A2:** How severe would you consider the consequences of the following hazards? (Please provide a numeric value in the interval provided.).

Severity of a Hazard for the Wrist and Elbow Rehabilitation System		Please Provide a Value between 0 (no Damages) and 100 (May Result in Death or Loss of System
M1	The arm of the patient may be crushed by the moving mechanism. This hazard may be created by incorrect posture of the patient, wrong parameters introduced in the control system (not suitable for patient movement capabilities), it can even be a consequences of a power failure	
M2	The patent may bruise or cut its hand in the edges of the components of the robotic structure. This may occur due to improper design of mechanical components of the robotic system.	
M3	Motion limit exceeded. The robot either exceeds the moving capabilities of the patient or the moving mechanism goes over the end stroke limiters.	
M4	The patient may crush into the robotic structure. Usually the patient is either carried using a wheelchair or if he can walk is often suspected by sudden loss of equilibrium.	
M5	The patient may have sudden spasms resulting in incontrollable motion of the body. The robot should be able to overcome and resist to this situation, allowing in the same time easy removal of the patient’s arm from the rehabilitation device.	
E1	Risk of electrocution of the patient.	
E2	Harming the patient due to sensor malfunction.	
E3	Crush the arm of the patient caused by the malfunction of stroke limiters.	
E4	Risk of short-circuit	
E5	Overloading the rehabilitation device	
T1	The patient may suffer burns by coming into contact with overheating parts of the robot.	
N1	Due to the fact that the robot works quite close to the patient, the sound created by moving mechanisms may create some discomfort for the patient.	
V1	Patient may be harmed by loose parts from the robotic system.	
V2	Patient may be harmed by uncontrolled vibrations of the mechanism.	
ER1	The patient may fall due to the fact that he is in a wheelchair.	
